# YouTube and ‘psychiatry’

**DOI:** 10.1192/pb.bp.114.050013

**Published:** 2015-12

**Authors:** Robert Gordon, John Miller, Noel Collins

**Affiliations:** 1 University Hospital Southampton NHS Foundation Trust, Southampton; 2 Universidad Complutense de Madrid, Spain (Masters student); 3 Surrey and Borders Partnership NHS Foundation Trust, Godalming

## Abstract

YouTube is a video-sharing website that is increasingly used to share and disseminate health-related information, particularly among younger people. There are reports that social media sites, such as YouTube, are being used to communicate an anti-psychiatry message but this has never been confirmed in any published analysis of YouTube clip content. This descriptive study revealed that the representation of ‘psychiatry’ during summer 2012 was predominantly negative. A subsequent smaller re-analysis suggests that the negative portrayal of ‘psychiatry’ on YouTube is a stable phenomenon. The significance of this and how it could be addressed are discussed.

YouTube (www.youtube.com) is a video-sharing website created in 2005, which now provides a platform for 2 billion clip viewings every day.^1^ The website's viewership statistics are eye opening. More than 1 billion unique users visit YouTube each month. Over 6 billion hours of video content are watched each month and 100 hours of video are uploaded to YouTube every minute. It is available in 61 countries and across 61 languages, and according to Nielsen, YouTube reaches more US adults aged 18-34 than any cable network.^2^

Clip content is varied, uncontrolled and often anonymous. Whereas clips are uploaded and accessed by diverse individuals, groups and political bodies across the globe, universal access is restricted in some countries. In China, for example, YouTube access was blocked by the government in 2009 as a response to Tibetan content.^3^ YouTube website administrators also reserve the right to remove uploaded content should users violate specified terms and conditions. Video files that ignite social political unrest, violate copyright and intellectual property protection laws, or breach national security legislation are likely to be removed. According to the YouTube terms of service, material which is obscene, defamatory or unlawful must not be submitted.^4^ Despite these restrictions, the YouTube platform is largely an unregulated medium which is being used to circulate extraordinarily varied material. This includes sharing and disseminating health-related information, particularly among younger people.^5,6^ Against this backdrop, there is growing concern that internet social media are being increasingly used to communicate an anti-psychiatry message.^7^ However, to our knowledge, this has never been confirmed in any published systematic analysis of YouTube content. This descriptive study aimed to determine whether YouTube portrayed ‘psychiatry’ in a positive, neutral or negative light. In view of the enormity of the viewership statistics, we consider the implications of this.

## Study

On 12 July 2012 we examined YouTube on default search settings using the solitary search term ‘psychiatry’. We believed this to be the most appropriate description of the discipline as a whole and the most likely reductionist YouTube search term for the specialty. Other possible search terms such as ‘mental health’ were discounted owing to the conceptual overlap with psychopathology and other constructs and the likelihood of generating broad and irrelevant search results. YouTube ranks search results according to relevance gained from the title of the clip, descriptive language within ‘relevant keywords’ and ‘video tags’, and the video description itself. Furthermore, the higher the number of ‘comments’ a video possesses, the higher the ‘authority signal’ becomes (i.e. the inherent video popularity, which also promotes a higher ranking).^8^

The first 100 clips of more than 1000 ranked results were viewed independently by two researchers (R.G. and J.M.) and categorised as positive, negative or neutral in their representation of psychiatry. No explicit criteria were used in categorising clips, which were assigned to each category according to the global impression of reviewer regarding the overall theme of each clip. Clips were assigned ‘neutral’ as a default if no overall negative or positive theme was identified. Disagreements in clip category were arbitrated by a third reviewer (N.C.). The number of views and clip length were also recorded. Non-functioning, deleted or repeated clips were excluded from analysis. Browser software at the time of examination was up to date and there was no disagreement between non-functioning clips observed on different computers.

## Study results

The kappa agreement between observers was 76%. The observers excluded 20 clips from analysis. The majority of eligible clips portrayed psychiatry negatively (51%) compared with neutrally (29%) and positively (20%) (Table 1). Negative clips were viewed more frequently and were longer than both positive and neutral clips. A subsequent smaller re-analysis of the first ten ranked clips on 14 July 2013 (five negative, two positive, three neutral) and 13 August 2014 (six negative, one positive, three neutral) revealed similar findings. A selection of analysed clips is provided in Box 1.

**Table 1:** Characteristics of negative, positive and neutral clips on 12 July 2012

Clip type	Eligible clips, *n* (%)	Average number of clips views	Average clip length
Negative	41 (51)	77 035	14m 49s

Positive	16 (20)	54 234	9m 34s

Neutral	23 (29)	7244	7m 20s

Common themes of negative clips related to the process of diagnosis and treatment. In particular, psychiatric diagnoses were criticised for being invalid, unreliable and a non-scientific mechanism of social control. Other concerns included stigma and the ‘labelling’ of individuals with diagnoses, administering toxic psychotropic medication to children and the potential harm of psychiatric treatment. Themes of positive clips included the benefits of psychiatric research, improvements in treatment and an anti-stigma video (‘Beards and Bowties’ by Dr Kamran Ahmed: http://www.youtube.com/watch?v=70loMcIqd9Q). Neutrally themed clips were largely educational in nature, including a cartoon portrayal of the proposed neurotransmitter mechanism of bipolar affective disorder.

## Discussion

This study reveals that the representation of ‘psychiatry’ on YouTube in July 2012 was predominantly negative. Subsequent clip analyses in 2013 and 2014 suggest that this is a stable phenomenon. The source of negative YouTube clips was unclear owing to blind authorship. However, there were three ‘regularly negative’ authors of clips promoting a seemingly ‘anti-psychiatry’ campaign.

What does a search term of ‘psychiatry’ mean? Content analysis of clips suggested the discipline of psychiatry itself was the implied meaning of ‘psychiatry’ in the majority of clips. Although other medical specialties also suffer from negative portrayals on YouTube, this is usually topic-specific such as paediatric immunisation or objections to tanning by dermatologists.^9-11^ ‘Psychiatry’ as a medical discipline appears uniquely targeted on YouTube for negative representation.

Why is portrayal of psychiatry on YouTube negative? The YouTube medium itself is vulnerable to extreme content owing to blind authorship, presentation of opinion as fact and the distinct lack of any peer review or editorial process.^3^ The online anti-psychiatry campaign has been linked to Scientology, disgruntled patients and psychiatrists, critical social scientists, humanistic psychologists and journalists sceptical towards psychiatry.^7^ It has also been suggested that an anti-psychiatry group now exists as a patient-based consumer movement.^7^ This online antipsychiatry message may be increasing,^7^ with the release of DSM-5 being a particular nidus of further criticism.^12^ The negative online representation of ‘psychiatry’ may also be an extension of long-standing societal scepticism of ‘psychiatry’ into a contemporary medium. It could also be symptomatic of the overall failure of psychiatry to promote itself more positively.

It is unclear whether information disseminated through social media platforms influences health-related attitudes and behaviours.^6^ More educated viewers appear relatively resistant to inaccurate information on YouTube, even when the message is framed as scientific reasoning.^6^ However, it does appear that social media websites are becoming an increasingly popular source of health information.^13^ The spiralling volume of uncensored information being uploaded to social video platforms such as YouTube makes it difficult for heath consumers to discern reliable health information from misleading content. Certain patient groups, such as younger adults and people with anorexia, may be more vulnerable to extreme content.^13^ It is also unclear how the negative online representation of psychiatry interacts with real-world stigma surrounding psychiatric illness and its treatment.

**Box 1** A selection of clips from the original 2012 analysis

**Negative:**

http://www.youtube.com/watch?v=y_AC-JhPOI (The psychiatric drugging of children & elderly)

http://www.youtube.com/watch?v=hy79C0v8elE (Psychiatry)

http://www.youtube.com/watch?v=PcuhhJ1BaMk (The DSM: psychiatry's deadliest scam)

**Positive:**

http://www.youtube.com/watch?v=dFs9WO2B8uI (RSA animate – the divided brain)

http://www.youtube.com/watch?v=tTCwihayOv0 (Peggy Rodriguez, MD for UNM Department of Psychiatry Residency Program)

http://www.youtube.com/watch?v=89-LDCnP8qw (Anthony Rothschild, MD: Brudnick Chair & professor of psychiatry)

**Neutral:**

http://www.youtube.com/watch?v=5N8LJjGjsfI (Ask the doctor: cardiology, psychiatry, geriatric medicine)

https://www.youtube.com/watch?v=qVkYHioCHpk (Psychiatry, Ain Shams University, basic interviewing skills 1.wmv)

https://www.youtube.com/watch?v=jq5F2XRt6QM (Psychiatrist vs psychologist (mental health guru))

Despite negative representation, viewing rates of YouTube ‘psychiatry clips’ are low compared with other content. The most popular ‘psychiatry’ clip, a music video by The Avalanches entitled ‘Frontier psychiatrist’, including a parody of the psychotherapist and patient encounter, attracted 2.3 million views. To give some perspective, ‘Gangham Style’, a music video by the South Korean musician Psy, attracted 1.5 billion views, ‘Charlie bit my finger again’ 520 million and the Taiwanese ‘Nyan cat’ animation 101 million views.

Although the effects of negative representations of psychiatry on social media remain questionable, it is clear that YouTube content is capable of exerting global impact. Sceptics of this need only heed the story of Sonya the slow loris. In 2009, Dmitry Sergeyev uploaded a video of her being tickled. Although illegal to have a captive slow loris as a pet outside of Russia, this single viral video has increased the illegal pet trade of these animals and has now led to the near extinction of the species.^14^

### Psychiatry fighting back

Accepting that negative representation of psychiatry on YouTube is a concern, how can it be addressed? Psychiatrists, their professional bodies and healthcare providers could start by recognising the influence of social media and its potential for disseminating health information, particularly in younger health consumers.^5^ Promisingly, the Royal College of Psychiatrists has launched its own YouTube channel (www.youtube.com/user/RCofPsychiatrists). This currently has low impact, with its introductory clip displaying a modest (2000) number of views, but it could eventually be a platform to provide unbiased and accurate information and to convey a positive message about psychiatry more generally. It may be as important to raise awareness among younger people and vulnerable patient groups about the trustworthiness of online information more generally.^13^ Others have suggested political leverage on YouTube to communicate more objective information^6^ or to carry explicit disclaimers when an extreme view is represented (in the same manner as television). Further options include the development of algorithms to automatically detect and filter extreme videos before they become popular.^13^ However, these proposals are somewhat at odds with the overarching YouTube ethos of free ‘self-broadcast’.
